# Variations in the Uptake of Active Surveillance for Prostate Cancer and Its Impact on Outcomes

**DOI:** 10.1016/j.euros.2023.04.006

**Published:** 2023-05-15

**Authors:** Mats S. Ahlberg, Hans Garmo, Lars Holmberg, Anna Bill-Axelson

**Affiliations:** a Department of Surgical Sciences, Uppsala University, Uppsala, Sweden; b Regional Cancer Center Uppsala/Örebro, Uppsala University Hospital, Uppsala, Sweden; c Translational Oncology & Urology Research (TOUR), School of Cancer and Pharmaceutical Sciences, King’s College London, London, UK

**Keywords:** Active surveillance, Prostate cancer, Outcomes, Overtreatment

## Abstract

**Background:**

Regional differences in active surveillance (AS) uptake for prostate cancer (PC) illustrate an inequality in treatment strategies.

**Objective:**

To examine the association between regional differences in AS uptake and transition to radical treatment, start of androgen deprivation therapy (ADT), watchful waiting, or death.

**Design, setting, and participants:**

A Swedish population-based cohort study was conducted including men in the National Prostate Cancer Register in Sweden with low-risk or favorable intermediate-risk PC, starting AS from January 1, 2007 and continuing till December 31, 2019.

**Intervention:**

Regional tradition of low, intermediate, or high proportions of immediate radical treatment.

**Outcomes measurements and statistical analysis:**

Probabilities of transition from AS to radical treatment, start of ADT, watchful waiting, or death from other causes were assessed.

**Results and limitations:**

We included 13 679 men. The median age was 66 yr, median PSA 5.1 ng/ml, and median follow-up 5.7 yr. Men from regions with a high AS uptake had a lower probability of transition to radical treatment (36%) than men from regions with a low AS uptake (40%; absolute difference 4.1%; 95% confidence interval [CI] 1.0–7.2), but not a higher probability of AS failure defined as the start of ADT (absolute difference 0.4%; 95% CI –0.7 to 1.4). There were no statistically significant differences in the probability of transition to watchful waiting or death from other causes. Limitations include uncertainty in the estimation of remaining lifetime and transition to watchful waiting.

**Conclusions:**

A regional tradition of a high AS uptake is associated with a lower probability of transition to radical treatment, but not with AS failure. A low AS uptake suggests overtreatment.

**Patient summary:**

There are considerable regional differences in active surveillance (AS) uptake for prostate cancer. This study compared the outcomes of AS in different regions and found no association between AS uptake and failure of AS; it suggests that a low AS uptake indicates overtreatment.

## Take Home Message

Men from regions with a high active surveillance (AS) uptake had a lower risk of transition from AS to radical treatment, but not a higher risk of AS failure. A low AS uptake suggests overtreatment.

## 1 Introduction

There are considerable regional differences in the uptake of active surveillance (AS) for men with low- and intermediate-risk prostate cancer (PC) reflecting disparities in treatment strategies depending on location [1], [2], [3]. It is not known whether these regional differences in the proportions of immediate radical treatment are associated with the outcome of AS.

The criteria for determining which men with PC are suitable for AS have evolved since its introduction in the 1990s, and the willingness to include men in AS has generally increased [4]. In Sweden, there has been a sharp rise in the use of AS in the past decade, and about 90% of men with low-risk PC now start AS. Acceptance of offering AS to men with favorable intermediate-risk PC is increasing, but the regional differences persists [5], [6], [7]. Ongoing cohort studies of AS have gradually expanded the inclusion criteria, and guidelines now recommend AS for selected men with favorable intermediate-risk PC in addition to men with low-risk PC, even though the safety of AS for intermediate-risk PC is debated [8], [9], [10], [11], [12], [13], [14], [15], [16], [17]. Some centers have a high proportion of men with low-risk and favorable intermediate-risk PC who undergo immediate radical treatment, while other centers are more liberal with AS and have a lower proportion of immediate radical treatment. The proportion of men with low-risk and favorable intermediate-risk PC who undergo immediate radical treatment represents a selection to AS uptake that varies regionally.

The aim of this study was to examine whether there was an association between regions with different proportions of immediate radical treatment, as a proxy for selection to AS uptake, and the long-term outcomes of AS in terms of transition from AS to radical treatment with curative intent, start of androgen deprivation therapy (ADT) as a sign of AS failure, watchful waiting, or death from other causes. We hypothesized that a high AS uptake would be associated with a higher probability of transition to radical treatment and AS failure than a low AS uptake.

## 2 Patients and methods

This cohort study was approved by the Swedish Ethical Review Authority (Dnr. 2021-07051-02).

### 2.1 Study population

The National Prostate Cancer Register (NPCR) of Sweden has a capture rate of 98% of PCs in Sweden compared with the Swedish Cancer Registry where all diagnosed cancers are registered by law [18]. NPCR is linked to several other national registers, including the Prescribed Drug Registry and the Cause of Death Registry, by the Swedish personal identity number, creating the Prostate Cancer data Base Sweden (PCBaSe) from where we extracted data [18]. We used similar inclusion criteria to the SPCG-17 and PRIAS studies for assessing eligibility for AS [19], [20]. We included all men who were registered in NPCR as starting AS from January 1, 2007 and continuing till December 31, 2019, with clinical T-stage 1 (cT1) or 2 (cT2) PC, prostate-specific antigen (PSA) below 15 ng/ml, PSA density ≤0.2 ng/ml/cc, and any amount of Gleason score 3 + 3 = 6 or 3 + 4 = 7 in <30% of the cores. For cT1a and cT1b tumors diagnosed by transurethral resection of the prostate, additional biopsies were mandatory. All cT2 tumors were included if these met the other criteria. Patients were followed until event or loss to follow-up, or until the end of 2019 with a maximal follow-up of 12 yr.

### 2.2 Exposure: regional tradition of immediate radical treatment

**Fig. 1:**
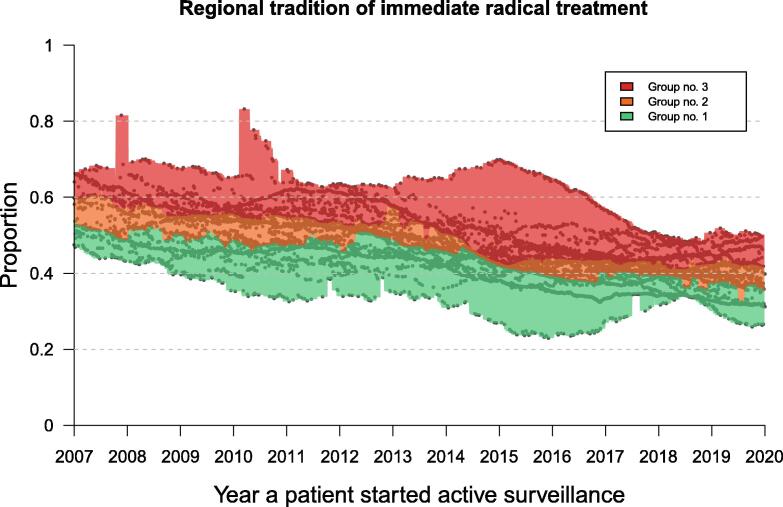
Regional tradition of immediate radical treatment defined as the proportion of men suitable for active surveillance (AS) who had undergone immediate radical treatment in the previous 3 yr, computed for every patient in the study at the start of AS. Based on the distribution of the regional tradition of immediate radical treatment, three equal-sized groups were defined: group 1 (green) with low-proportion immediate radical treatment, group 2 (yellow) with intermediate-proportion immediate radical treatment, and group 3 (red) with high-proportion immediate radical treatment. Every dot in the diagram represents a patient in the study starting AS.

For every man who started AS, we estimated a regional tradition of immediate radical treatment as the proportion of men suitable for AS (cT1 or cT2, PSA <15 ng/ml and Gleason score 3 + 3 = 6 or 3 + 4 = 7) in that specific health care region who had undergone immediate radical treatment in the previous 3 yr. Based on the distribution of the regional traditions of immediate radical treatment for all patients starting AS, we defined three equal-sized groups (tertiles) each year, based on the proportion of immediate radical treatment: group 1—low-proportion immediate radical treatment; group 2—intermediate-proportion immediate radical treatment; and group 3—high-proportion immediate radical treatment (Fig. 1).

We further explored whether the addition of the proportion of early transition from AS to radical treatment affected the results. For every man who remained in AS after 3 yr, we estimated the regional tradition of transition from AS to radical treatment in the first 3 yr of AS as the proportion of men who started AS in that specific health care region and who had transitioned to radical treatment the in first 3 yr of AS. We combined the proportion of immediate radical treatment with the proportion of transition from AS to radical treatment in the first 3 yr of AS for a sensitivity analysis.

### 2.3 Outcomes: probability of transition from active surveillance

The endpoints were transition from AS to radical treatment, start of ADT, watchful waiting, and death from other causes than PC. To estimate the transition to watchful waiting, we used a statistical model that estimates life expectancy [21]. The model is based on Charlson comorbidity index (CCI), a validated drug comorbidity index, and age, and all data were available in PCBaSe [22], [23]. When the estimated remaining lifetime, according to the model, dropped below 10 yr, men were considered to transition from AS to watchful waiting. We considered the transition from AS to the start of ADT as AS failure.

### 2.4 Statistical analyses

Continuous data are presented as medians and interquartile ranges, and categorical data as numbers and percentages. Follow-up time was quantified using the reverse Kaplan-Meier estimate of potential follow-up [24], [25]. We analyzed the association between the three groups with low (group 1), intermediate (group 2), and high (group 3) proportions of immediate radical treatment and the probability of transition from AS to radical treatment, start of ADT, watchful waiting, and death from other causes. We made a sensitivity analysis of the added effect of the regional tradition of low, intermediate, and high proportions of transition from AS to radical treatment in the first 3 yr of AS, for men who remained in AS after 3 yr.

Probabilities of transition from AS were estimated as cumulative incidence proportions with 95% confidence intervals (CIs) and censored at the last day of follow-up [26]. We primarily wanted to assess group differences between group 1 and group 3. Group differences were estimated with both relative risks as hazard ratios (HRs) using Cox regression, with group 1 as reference group (HR = 1), and as absolute differences at 12 yr of AS. Results are presented with 95% CI. HRs were estimated from both an unadjusted and an adjusted model for potential confounders (age, PSA, PSA density, risk group, Gleason score, cT stage, number of biopsies with cancer, millimeters of cancer in biopsies, and CCI).

## 3 Results

In all, 13 679 men fulfilled the inclusion criteria and were included in the study. The median age was 66 yr at the start of AS. The median PSA was 5.1 ng/ml, median PSA density 0.12 ng/ml/cc, and median follow-up time 5.7 yr. Eighty-five percent had low-risk PC, 90% had Gleason score 3 + 3 = 6 or less, and 84% had cT1 tumors. The figures for the three groups with a tradition of low-, intermediate-, and high-proportion immediate radical treatment were similar. Of all men who started AS, 59% remained in AS after 3 yr (Table 1). The regional proportion of immediate radical treatment varied between 43% and 82% in 2007 and between 26% and 52% in 2019 (Fig. 1).

**Table 1:** Basic characteristics for all patients in the study, and presented separately for group 1 (low-proportion immediate radical treatment), group 2 (intermediate-proportion immediate radical treatment), and group 3 (high-proportion immediate radical treatment), and for patients remaining in active surveillance (AS) after 3 yr

	All patients	Low-proportion immediate radical treatment	Intermediate-proportion immediate radical treatment	High-proportion immediate radical treatment	Patients remaining in AS after 3 yr
	*n* = 13 679	*n* = 4566 (group 1)	*n* = 4562 (group 2)	*n* = 4551 (group 3)	*n* = 8013
Age (yr), median (IQR)	65.9 (61.3–69.6)	66.1 (61.5–69.7)	66.0 (61.4–69.5)	65.7 (61.2–69.6)	65.5 (61.0–68.9)
PSA (ng/ml), median (IQR)	5.1 (4.0–6.8)	5.3 (4.1–7.0)	5.0 (3.9–6.6)	5.1 (4.0–6.7)	5.0 (3.9–6.7)
PSA density (ng/ml^2^), median (IQR)	0.12 (0.09–0.15)	0.12 (0.09–0.15)	0.12 (0.09–0.15)	0.12 (0.09–0.15)	0.12 (0.09–0.14)
Follow-up time (yr), median (IQR)	5.7 (3.4–8.4)	5.6 (3.4–8.3)	5.8 (3.4–8.5)	6.1 (3.4–8.3)	6.5 (5.0–9.2)
Prostate cancer risk group, no. (%)					
Low risk	11 681 (85)	3874 (85)	3920 (86)	3887 (85)	7045 (88)
Intermediate risk	1998 (15)	692 (15)	642 (14)	664 (15)	968 (12)
Gleason score, no. (%)					
≤3 + 3 = 6	12 348 (90)	4106 (90)	4129 (91)	4113 (90)	7426 (93)
3 + 4 = 7	1331 (10)	460 (10)	433 (9)	438 (10)	587 (7)
cT stage, no. (%)					
1a	55 (0.4)	23 (0.5)	18 (0.4)	14 (0.3)	33 (0.04)
1b	24 (0.2)	10 (0.2)	6 (0.1)	8 (0.2)	14 (0.02)
1c	11 522 (84)	3846 (84)	3825 (84)	3851 (85)	6977 (87)
2	2078 (15)	687 (15)	713 (16)	678 (15)	989 (12)
No. of positive bx cores, no. (%)					
1	8341 (61)	2671 (58)	2795 (61)	2875 (63)	5195 (65)
2	4180 (31)	1442 (32)	1429 (31)	1309 (29)	2279 (28)
3	1115 (8.2)	432 (9)	328 (7)	355 (8)	514 (6.4)
4	40 (0.3)	21 (0.5)	19 (0.2)	9 (0.2)	23 (0.3)
5	3 (0.02)	0 (0)	0 (0)	3 (0.07)	2 (0.02)
mm cancer in bx, median (IQR)	2 (1–3.5)	2 (1.0–4.0)	2 (1.0–3.5)	2 (1.0–3.1)	1.7 (1.0–3.0)
Missing, no. (%)	1156 (8.6)	409 (9.0)	364 (8.0)	383 (8.4)	781 (9.7)
CCI, no. (%)					
0	11 857 (87)	3933 (86)	3990 (87)	3934 (86)	7048 (88)
1	1095 (8.0)	356 (8)	369 (8)	370 (8)	594 (7.4)
2	616 (4.5)	240 (5)	169 (5)	207 (5)	319 (4.0)
3	96 (0.7)	34 (0.7)	27 (0.6)	35 (0.7)	47 (0.6)
4	15 (0.1)	3 (0.07)	7 (0.2)	5 (0.1)	5 (0.06)

AS = active surveillance; bx = biopsy; CCI = Charlson Comorbidity Index; cT stage = clinical T stage; IQR = interquartile range; PSA = prostate-specific antigen.

After 12 yr of follow-up, in the whole group, the probabilities of transition from AS to radical treatment, start of ADT, watchful waiting, and death from other causes were 39%, 3.6%, 27%, and 3.6%, respectively.

**Fig. 2:**
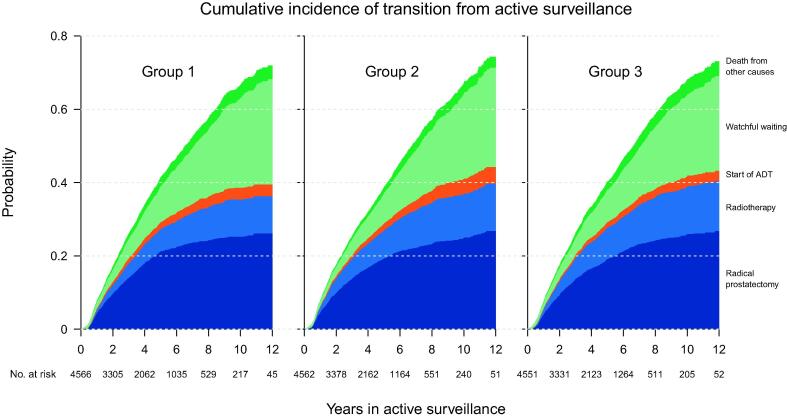
Cumulative incidence of transition from active surveillance to radical treatment (presented as radical prostatectomy and radiotherapy separately), start of androgen deprivation therapy (ADT), transition to watchful waiting, and death form other causes for group 1 (low proportion immediate radical treatment), group 2 (intermediate proportion immediate radical treatment), and group 3 (high proportion immediate radical treatment).

The probability of transition to radical treatment was 36% in group 1 and higher in group 2 (40%) and group 3 (40%) with a higher proportion of immediate radical treatment. The absolute difference between groups 1 and 3 was 4.1% (95% CI 1.0–7.2). For transition to ADT, the probabilities were 3.2%, 4.6%, and 2.8% for groups 1, 2, and 3, respectively, but the small absolute difference of 0.4% between groups 1 and 3 was not significant. The probability of transition to watchful waiting decreased with increasing proportions of immediate radical treatment, and the probabilities were 29%, 27%, and 26% for groups 1, 2, and 3, respectively, but the absolute difference of 3.0% between groups 1 and 3 was not significant. The probabilities of death from other causes were 3.8%, 3.0%, and 4.1 % for groups 1, 2, and 3, respectively, but the difference between groups 1 and 3 was not significant (Fig. 2 and Table 2).

**Table 2:** Probability of transition from active surveillance.

Proportion immediate radical treatment:	Probability of transition to:					
	All radical treatment		Radical prostatectomy		Radiotherapy	
	%	95% CI	%	95% CI	%	95% CI
Low *(Group 1)*	36.2	34.2–38.3	26.1	24.3–27.9	10.1	8.9–11.4
Intermediate *(Group 2)*	39.7	37.3–42.0	26.8	24.8–28.8	12.9	11.4–14.4
High *(Group 3)*	40.4	38.0–42.7	26.8	24.8–28.8	13.6	12.1–15.1
All patients	38.8	37.5–40.2	26.6	25.5–27.7	12.2	11.4–13.1
**Absolute differences:**						
Low vs. intermediate	3.4	0.3–6.6	0.7	−2.1–3.4	2.8	0.8–4.7
Low vs. high	4.1	1.0–7.2	0.7	−2.0–3.4	3.4	1.5–5.4
Intermediate vs. high	0.7	−2.6–4.0	0.03	−2.8–2.9	0.7	−1.5–2.8

**Proportion immediate radical treatment:**	**Probability of transition to:**					

	**ADT**		**Watchful waiting**		**Death from other causes**	
	%	95% CI	%	95% CI	%	95% CI

Low *(Group 1)*	3.2	2.5–3.9	28.7	25.9–31.6	3.8	3.0–4.6
Intermediate *(Group 2)*	4.6	3.5–5.6	27.1	24.5–29.7	3.0	2.3–3.7
High *(Group 3)*	2.8	2.1–3.6	25.8	23.2–28.4	4.1	3.2–5.0
All patients	3.6	3.1–4.1	27.2	25.7–28.7	3.6	3.2–4.1
Absolute differences:						
Low vs. intermediate	1.4	0.1–2.6	1.7	−2.2–5.5	0.8	−0.3–1.8
Low vs. high	0.4	−0.7–1.4	3.0	−0.9–6.8	0.3	−0.8–1.5
Intermediate vs. high	1.7	0.5–3.0	1.3	−2.4–5.0	1.1	0.0–2.2

Probability of transition from active surveillance (AS) to radical treatment (and presented as radical prostatectomy and radiotherapy separately), start of androgen deprivation therapy (ADT), transition to watchful waiting, and death from other causes than prostate cancer after 12 years of AS for all patients and group 1 (low proportion immediate radical treatment), group 2 intermediate proportion immediate radical treatment) and group 3 (high proportion immediate radical treatment). Absolute differences between groups are presented with 95% confidence intervals.

When analyzing the two methods of radical treatment separately, the probability of transition to radical prostatectomy was similar in the three groups (26%, 27%, and 27% for groups 1, 2, and 3, respectively). For transition to radiotherapy, we found the same trend as we observed for transition to all radical treatments, with an increasing probability of transition to treatment with an increasing proportion of immediate radical treatment. The probabilities of transition to radiotherapy were 10%, 13%, and 14% for groups 1, 2, and 3, respectively, with an absolute difference between groups 1 and 3 of 3.4% (95% CI 1.5–5.4; Fig. 2 and Table 2).

In the unadjusted Cox regression model, there was no difference in the probability of transition to radical treatment between group 3 and the reference group, but in the adjusted Cox regression model, group 3 had a higher probability of transition to radical treatment than the reference group (HR 1.09; 95% CI 1.0–1.2). There were no significant differences for the start of ADT, and transition to watchful waiting or death from other causes in the unadjusted or adjusted model between group 3 and the reference group. When analyzing the probability of transition to radical prostatectomy and radiotherapy separately, we found no differences for transition to radical prostatectomy, but for transition to radiotherapy, group 3 had a higher probability than the reference group in the unadjusted (HR 1.3; 95% CI 1.1–1.6) and adjusted (adjusted HR 1.4; 95% CI 1.2–1.6) models (Table 3).

**Table 3:** Hazard ratios for transition from active surveillance.

	Transition to all radical treatment				Transition to radical prostatectomy				Transition to radiotherapy			
Proportion immediate radical treatment:	HR	95%CI	aHR	95%CI	HR	95%CI	aHR	95%CI	HR	95%CI	aHR	95%CI
Low *(Group 1)*	1		1		1		1		1		1	
Intermediate *(Group 2)*	1.03	0.95–1.12	1.08	0.99–1.18	0.96	0.87–1.05	0.99	0.90–1.10	1.26	1.08–1.46	1.34	1.14–1.58
High *(Group 3)*	1.07	0.98–1.15	1.09	1.00–1.19	0.97	0.88–1.07	1.00	0.90–1.10	1.34	1.16–1.56	1.37	1.17–1.61

	**Start of ADT**				**Transition to watchful waiting**				**Transition to radiotherapy**			
**Proportion immediate radical treatment:**	**HR**	**95%CI**	**aHR**	**95%CI**	**HR**	**95%CI**	**aHR**	**95%CI**	**HR**	**95%CI**	**aHR**	**95%CI**

Low *(Group 1)*	1		1		1		1		1		1	
Intermediate *(Group 2)*	1.10	0.84–1.46	1.17	0.86–1.60	0.88	0.79–0.99	0.91	0.79–1.04	0.77	0.58–1.02	0.75	0.56–1.01
High *(Group 3)*	0.81	0.60–1.10	0.82	0.59–1.16	0.91	0.81–1.02	0.91	0.80–1.04	1.02	0.79–1.32	1.06	0.81–1.39

Unadjusted and adjusted hazard ratios (HR/aHR) for transition from active surveillance (AS) to radical treatment (and presented as radical prostatectomy and radiotherapy separately), start of androgen deprivation therapy (ADT), and death from other causes until 12 years of AS. Group 1 with regional tradition of low proportion immediate radical treatment is index group (HR = 1).

### 3.1 Sensitivity analysis

A sensitivity analysis of the added effect of the regional tradition of low-, intermediate-, and high-proportion transition from AS to radical treatment in the first 3 yr of AS did not change the main results (Supplementary Tables 1 and 2).

## 4 Discussion

In this population-based study, we found that men from regions with a high AS uptake had a slightly lower probability of transition from AS to radical treatment than men from regions with a low AS uptake, but not a higher probability of AS failure. We found no significant difference in the probability of transition from AS to watchful waiting or death from other causes between groups.

A high proportion of immediate radical treatment for men deemed suitable for AS implies a narrow selection of the most suitable patients starting AS, and we expected a low probability of transition to radical treatment, a low probability of AS failure, and a higher probability of transition to watchful waiting in that group. On the contrary, a low proportion of immediate radical treatment indicates a wide selection for AS, with an expected higher probability of transition to radical treatment, a higher probability of AS failure, and a lower probability of transition to watchful waiting. In contrast to our hypothesis, we found that what seems to be a regionally bound lower preference for radical treatment before starting AS continues to be a lower preference for transition from AS to radical treatment, but the probability of AS failure in this group, defined as the start of ADT, was not higher. Simultaneously, a higher preference for radical treatment before starting AS continues to be a higher preference for transition from AS to radical treatment. The differences in transition to radical treatment between the groups are small and the clinical relevance is reasonably minor, but it does not seem that a higher preference for radical treatment is protective of AS failure, but rather suggests overtreatment.

After 5 yr of AS, the probabilities of transition to radical treatment and watchful waiting have been reported to be around 35–40% and <5%, respectively [8], [14], [27], [28]. We found a lower probability of transition to radical treatment, which might partly be explained by the coincident considerably higher probability of transition to watchful waiting (Fig. 2 and Table 2). Differences may also be due to local traditions of transition to radical treatment, as triggers are not specified in guidelines but rather left for the treating urologist and patient to decide together [10], [11], [12], [29].

Our finding of a high probability of transition to watchful waiting, which was similar in the three groups, indicates that AS was equally successful in all groups. The transition from AS to watchful waiting is recommended when life expectancy reaches below 10 yr and men are deemed not to benefit from treatment with curative intent anymore. It is not considered an AS failure but rather a success. In the large AS cohorts of GAP3 and PRIAS, the transition from AS to watchful waiting is assessed based on reports from each including center and, after 5 yr of AS, is found to be only a few percent, at the most [8], [28]. This report is subjective and based on respective clinicians’ assessment of the patient, as always in clinical practice, and even experienced clinicians have difficulty accurately estimating 10-yr life expectancy in patients [30].

The transition from AS to watchful waiting is rarely documented in medical charts and is not available in any registries, making it difficult to evaluate. Even if some patients who transitioned from AS to the start of ADT had already transitioned to watchful waiting before starting ADT, registry data cannot make this assumption and they are registered as AS failure in this study. To generate a more accurate estimate of the probability of events for men in AS, we assessed the transition to watchful waiting using a statistical model that estimates the expected remaining lifetime [21]. Evaluation of the model has shown a high grade of accurate estimation of expected remaining lifetime compared with age and CCI alone [23]. As we found much higher probabilities of transition to watchful waiting compared with earlier AS studies, the statistical model might have underestimated the expected remaining lifetime compared with the clinical evaluation of the patient and thus led to an overestimation of the probability of transition to watchful waiting. However, it could also imply that, in earlier AS studies, a non-negligible number of patients transitioned to radical treatment even though watchful waiting was a more adequate choice of strategy due to life expectancy of <10 yr.

In this study of men with a median age of 66 yr at the start of AS, the probability of dying from other causes than PC within 12 yr was 3.6%, which is comparable with what is expected in the general population [31]. The proportion of men dying from other causes increased gradually throughout follow-up, which reflects the real-world setting of the register. The risk of transition from AS to death from PC is negligible, as it is very unlikely that a man dies from PC without first starting ADT or transition to watchful waiting.

For men with Gleason score 3 + 4 = 7, we limited the inclusion to men with <30% positive biopsy cores, similar to the definitions in SPCG17 and PRIAS, where multiple positive targeted biopsy cores from the same lesion are considered one positive core [19], [20]. In NPCR, all positive biopsy cores, including targeted biopsies from the same lesion, are registered. This probably has led to the exclusion of some patients with >30% of cores with Gleason score 3 + 4 = 7 due to an overestimation of the number of positive cores, but there was no difference between the groups. In addition, before 2015, magnetic resonance imaging and targeted biopsies for PC were not in clinical practice in Sweden, and most patients in our cohort were diagnosed solely by systematic biopsies.

### 4.1 Strengths and limitations

The validity and completeness of NPCR have been assessed and found to be high [18]. The large cohort provides a high level of statistical precision. Compared with other studies of AS, we have been able to assess the transition from AS to watchful waiting in a unique way, which strengthens the internal validity of our study. Although the model that we used to estimate the remaining lifetime is validated as highly accurate, it is not a direct description of how patients were assessed by clinicians and real-life practice is unknown. Additionally, we do not have data on PSA relapse, adverse pathology, metastatic disease, or death from PC after radical treatment that could be considered AS failure. The follow-up time is also too short to detect late signs of AS failure. However, the distribution of known risk factors for AS failure (Gleason score, cT stage, and risk group) in the three groups was similar, which decreases the risk of a difference in unconsidered AS failure between the groups. In addition, in the long-term follow-up of AS cohorts, with similar characteristics to those of the cohort in this study, the oncological outcomes are excellent and late failures uncommon [9].

## 5 Conclusions

A regional tradition of a low proportion of immediate radical treatment and a high AS uptake are not associated with worse outcomes of AS. A high proportion of immediate radical treatment and early transition from AS to radical treatment suggest overtreatment.

  ***Author contributions:*** Mats S. Ahlberg had full access to all the data in the study and takes responsibility for the integrity of the data and the accuracy of the data analysis.

*Study concept and design*: Ahlberg, Garmo, Holmberg, Bill-Axelson.

*Acquisition of data*: Ahlberg, Garmo.

*Analysis and interpretation of data*: Ahlberg, Garmo, Holmberg, Bill-Axelson.

*Drafting of the manuscript*: Ahlberg, Bill-Axelson.

*Critical revision of the manuscript for important intellectual content*: Ahlberg, Bill-Axelson, Holmberg.

*Statistical analysis*: Ahlberg, Garmo.

*Obtaining funding*: Bill-Axelson, Ahlberg.

*Administrative, technical, or material support*: Bill-Axelson, Garmo.

*Supervision*: Bill-Axelson, Garmo, Holmberg.

*Other*: None.

  ***Financial disclosures:*** Mats S. Ahlberg certifies that all conflicts of interest, including specific financial interests and relationships and affiliations relevant to the subject matter or materials discussed in the manuscript (eg, employment/affiliation, grants or funding, consultancies, honoraria, stock ownership or options, expert testimony, royalties, or patents filed, received, or pending), are the following: None.

  ***Funding/Support and role of the sponsor:*** This study was supported by Swedish Cancer Society (grant 190020), the Swedish Prostate Cancer Society (grant number: [NA]), the Percy Falk Foundation (grant number: NA), the Foundation of the memory of Johanna Hagstrand and Sigfrid Linnér (grant number: NA), and the Hillevi Fries Foundation (grant number: NA). The funders of the study had no role in the conduct, data collection, management, analysis, and interpretation of the study; preparation or approval of the manuscript; or the decision to submit for publication.

  ***Acknowledgments:*** This project was made possible by the continuous work of the National Prostate Cancer Register of Sweden Steering Group: David Robinson (register holder), Ingela Franck Lissbrant (chairman), Johan Styrke (co-chairman), Johan Stranne, Jon Kindblom, Camilla Thellenberg, Andreas Josefsson, Ingrida Verbiené, Hampus Nugin, Stefan Carlsson, Anna Kristiansen, Karin Holmsten, Mats Andén, Thomas Jiborn, Olof Akre, Per Fransson, Eva Johansson, Johan Stranne, Magnus Törnblom, Fredrik Jäderling, Marie Hjälm Eriksson, Lotta Renström Koskela, Jonas Hugosson, Ola Bratt, Erik Thimansson, Jens Sörensen, Maria Nyberg, Fredrik Sandin, Marie Brus, Gustaf Hedström Anna Hedström, Nina Hageman, Christofer Lagerros, Hans Joelsson, Pär Stattin, and Gert Malmberg. These individuals were not compensated financially for their contributions.
