# Supplementary material for: Variations in the Uptake of Active Surveillance for Prostate Cancer and Its Impact on Outcomes
Source: Eur Urol Open Sci. 2023 May 15;52:166–73. doi: 10.1016/j.euros.2023.04.006 (PMC10240510; doi:10.1016/j.euros.2023.04.006)
Supplement: Supplementary data 1 [file mmc1.docx]

| Supplementary Table 1. Probability of transition from active surveillance | | | | | | |
| --- | --- | --- | --- | --- | --- | --- |
|  | | | | | | |
| Combined proportion radical treatment: | **Probability of transition to:** | | | | | |
|  | All radical treatment | | Radical prostatectomy | | Radiotherapy | |
|  | % | 95% CI | % | 95% CI | % | 95% CI |
| Low (*Group 1)* | 24.3 | 21.5-27.2 | 15.5 | 13.2-17.9 | 8.8 | 7.0-10.6 |
| Intermediate (*Group 2)* | 28.8 | 26.1-31.4 | 18.9 | 16.6-21.1 | 9.9 | 8.2-11.6 |
| High (*Group 3)* | 27.5 | 24.6-30.5 | 17.0 | 14.6-19.4 | 10.5 | 8.6-12.4 |
|  |  |  |  |  |  |  |
| All patients | 27.1 | 25.5-28.7 | 17.3 | 15.9-18.7 | 9.8 | 8.8-10.8 |
|  |  |  |  |  |  |  |
| Absolute differences: |  |  |  |  |  |  |
| Low vs. intermediate | 4.4 | 0.5-8.3 | 3.3 | 0.0-6.6 | 1.1 | -1.4-3.5 |
| Low vs. high | 3.2 | -0.9-7.3 | 1.5 | -1.9-4.9 | 1.7 | -0.9-4.3 |
| Intermediate vs. high | 1.2 | -2.7-5.2 | 1.9 | -1.5-5.2 | 0.6 | -1.9-3.1 |
|  | | | | | | |
| Combined proportion radical treatment: | **Probability of transition to:** | | | | | |
|  | ADT | | Watchful waiting | | Death from other causes | |
|  | % | 95% CI | % | 95% CI | % | 95% CI |
| Low (*Group 1)* | 3.0 | 2.0-4.1 | 33.4 | 29.4-37.4 | 3.2 | 2.1-4.3 |
| Intermediate (*Group 2)* | 3.9 | 2.7-5.1 | 27.5 | 24.6-30.4 | 3.0 | 2.2-3.9 |
| High (*Group 3)* | 3.2 | 2.2-4.1 | 30.0 | 26.3-33.6 | 3.3 | 2.3-4.3 |
|  |  |  |  |  |  |  |
| All patients | 3.4 | 2.8-4.0 | 30.2 | 28.2-32.2 | 3.2 | 2.6-3.7 |
|  |  |  |  |  |  |  |
| Absolute differences: |  |  |  |  |  |  |
| Low vs. intermediate | 0.9 | -0.7-2.5 | 5.9 | 1.0-10.8 | 0.2 | -1.2-1.6 |
| Low vs. high | 0.1 | -1.3-1.5 | 3.5 | -1.9-8.9 | 0.0 | -1.4-1.5 |
| Intermediate vs. high | 0.8 | -0.8-2.3 | 2.4 | -2.2-7.1 | 0.2 | -1.0-1.5 |

**Supplementary Table 1.** Probability of transition to radical treatment (and presented as radical prostatectomy and radiotherapy separately), start of ADT, transition to watchful waiting, and death from other causes than prostate cancer after 12 years of AS for all patients remaining is AS after three years and presented separately for groups with a tradition of low, intermediate and high combined proportion radical treatment. Absolute differences between groups are presented with 95% confidence intervals.

| Supplementary Table 2. Hazard ratios for transition from active surveillance | | | | | | | | | | | | | | | |
| --- | --- | --- | --- | --- | --- | --- | --- | --- | --- | --- | --- | --- | --- | --- | --- |
|  | | | | | | | | | | | | | | | |
| Combined proportion radical treatment: | Transition to all radical treatment | | | | | Transition to radical prostatectomy | | | | | Transition to radiotherapy | | | | |
|  | HR | 95%CI |  | aHR | 95%CI | HR | 95%CI |  | aHR | 95%CI | HR | 95%CI |  | aHR | 95%CI |
| Low *(Group 1)* | 1 |  |  | 1 |  | 1 |  |  | 1 |  | 1 |  |  | 1 |  |
| Intermediate *(Group 1)* | 1.16 | 1.02-1.33 |  | 1.11 | 0.96-1.28 | 1.17 | 0.99-1.38 |  | 1.09 | 0.91-1.30 | 1.15 | 0.91-1.45 |  | 1.14 | 0.88-1.47 |
| High *(Group 1)* | 1.13 | 0.98-1.30 |  | 1.13 | 0.97-1.31 | 1.06 | 0.89-1.27 |  | 1.05 | 0.87-1.26 | 1.26 | 1.00-1.61 |  | 1.31 | 1.01-1.69 |
|  |  |  |  |  |  |  |  |  |  |  |  |  |  |  |  |
| Combined proportion radical treatment: | Start of ADT | | | | | Transition to watchful waiting | | | | | Death from other causes | | | | |
|  | HR | 95%CI |  | aHR | 95%CI | HR | 95%CI |  | aHR | 95%CI | HR | 95%CI |  | aHR | 95%CI |
| Low *(Group 1)* | 1 |  |  | 1 |  | 1 |  |  | 1 |  | 1 |  |  | 1 |  |
| Intermediate *(Group 1)* | 1.18 | 0.78-1.79 |  | 1.09 | 0.66-1.80 | 0.92 | 0.80-1.07 |  | 0.99 | 0.84-1.18 | 1.05 | 0.71-1.55 |  | 1.09 | 0.71-1.67 |
| High *(Group 1)* | 1.27 | 0.83-1.94 |  | 1.44 | 0.88-2.36 | 0.93 | 0.80-1.08 |  | 0.96 | 0.80-1.15 | 1.09 | 0.73-1.63 |  | 1.15 | 0.74-1.77 |

**Supplementary Table 2.** Unadjusted and adjusted hazard ratios (HR/aHR) for transition from active surveillance (AS) to all radical treatment (and presented as radical prostatectomy and radiotherapy separately), start of androgen deprivation therapy (ADT), and death from other causes for patients who remained in AS after three years. The group with low combined proportion early radical treatment is index group (HR=1). Adjustments were made for age, Prostate Specific Antigen (PSA), PSA-density, risk-group, Gleason score, clinical T-stage, number of biopsies with cancer and Charlson comorbidity index.
